# Imine-Based Reactive Mesogen and Its Corresponding Exchangeable Liquid Crystal Elastomer

**DOI:** 10.1021/acs.macromol.1c02432

**Published:** 2022-01-21

**Authors:** Xueyan Lin, Alexandra Gablier, Eugene M. Terentjev

**Affiliations:** Cavendish Laboratory, University of Cambridge, JJ Thomson Avenue, Cambridge CB3 0HE, United Kingdom

## Abstract

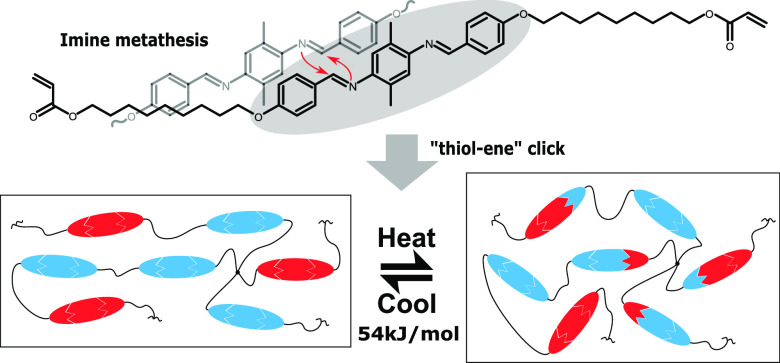

To date, exchangeable liquid crystalline elastomers (xLCEs) have been mainly fabricated by combining conventional LCEs with additional exchangeable functional groups in their networks. While conventional LCEs are frequently made from commercially available aromatic–ester reacting mesogens or from mesogens based on a biphenyl core, such reacting monomers are not optimized to fabricating xLCEs whose bond-exchange reaction is fast and clean cut. Here, we develop a fast synthesis route to produce a new type of reactive mesogen based on an aromatic–imine structure that intrinsically enables a fast and stable bond-exchange reaction in the resulting imine-based xLCE. This new xLCE displays vitrimer plastic-flow behavior, and its bond-exchange activation energy is calculated to be 54 kJ/mol. We also demonstrate that this xLCE is thermally stable to withstand many recycling cycles without visible decay, and its liquid crystallinity is preserved. Finally, we demonstrate the reprogramming and realignment of the mesogen orientation in this xLCE with the realigned xLCE capable of reversible thermal actuation.

## Introduction

Liquid crystalline elastomers (LCEs) are a class of functional materials that can be used as soft actuators.^1,2^ However, due to their cross-linked thermoset nature, it is often difficult to fabricate LCEs into complex geometries with their mesogen orientation specifically aligned to achieve sophisticated actuation patterns. Exchangeable LCEs (xLCE) offer a solution because their bond-exchange reactions can induce polymer network reconfiguration, which could generate a local anisotropy to guide mesogen alignment in the nematic state and thus allow fabrication of xLCEs of complex geometries with mesogens realigned.^3^ Out of the multiple bonds-exchange reactions discovered to date, only a fraction of reactions are exploited in the making of xLCEs by placing the functional groups outside of the mesogen molecule in the network.^4,5^ These reactions are transesterification, boronic ester exchange, carbamate exchange, siloxane exchange, disulfide exchange, Diels–Alder reaction, and cycloadditions, which are discussed in the cited reviews. However, all of these attractive and promising methods also have their limitations, which we will discuss now.

Transesterification is by far the most common strategy of bond exchange in xLCEs, but in general, it requires a large amount of catalyst and a high temperature to be activated.^6−10^ Recently a “catalyst-free” transesterification xLCE system was reported, achieved by incorporating the catalytic moiety into the network.^11^ However, transesterification in xLCE has been shown to damage the ester-based reactive mesogen (such as the popular RM257 and RM82) through an excessive reaction at high temperature, and the liquid crystalline phase can be destroyed as a consequence.^12^ This limits transesterification to the use of ester-free (e.g. biphenyl) mesogens. Boronic ester exchange requires no catalyst, and it is a fast exchange reaction that has been used in a room-temperature self-healing material.^13^ Unfortunately, due to its fast exchange rate, such xLCEs show some creep even at ambient temperatures and often require the aid of partial permanent cross-linking for mechanical stability.^14^ For carbamate exchange, a catalyst or extra hydroxyl groups are always required in the network to facilitate the exchange reaction.^15^ Here, possible catalyst degradation complicates the system, and the dissociative nature of the carbamate bonds indicates that the dissociated isocyanate group can be susceptible to water attack at high temperature.^16−18^ Disulfide exchange is also a fast reaction that does not require any catalyst, and the system also benefits from the radical-assisted dissociative mechanism.^19−21^ Nevertheless, in the past, we found that this type of xLCE suffers from oxidative side reaction at temperatures above 170 °C with sample tarring, which prevents the xLCE from multiple recycling. An xLCE based on the Diels–Alder reaction has advantages of being solution-cast and melt-drawn into useful geometries, but the system demands a long recovery time (up to 5 h) to reconnect the dissociated bonds, which creates practical problems.^22^ A [4 + 4] cycloaddition uses photopolymerizable moieties to reversibly cross-link xLCEs, but the synthesis of such photopolymerizable moieties can be challenging, and the network always requires prolonged light exposure to exchange due to light penetration issues.^23^

**Figure 1:**
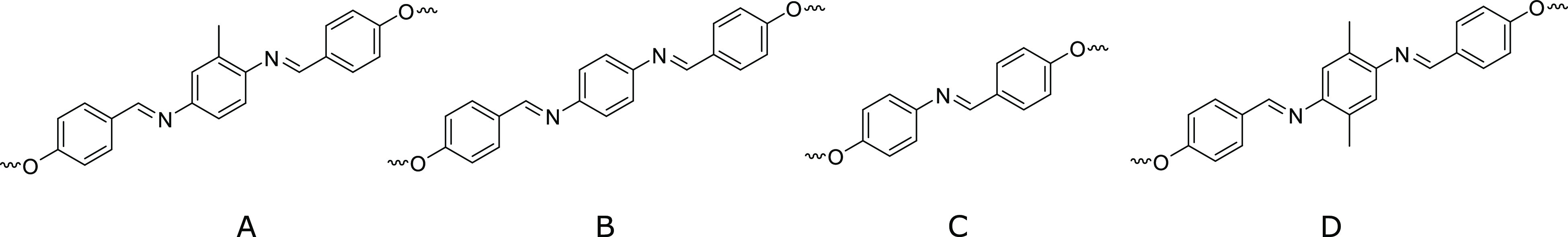
Mesogen core structures envisaged in our initial investigation. Starting chemical for structure A is difficult to handle due to air oxidation. Structure B gives mesogen that has poor solubility in our preferred solvents. Structure C has less-than-desired aspect ratio, and low mesogenic power. Our final choice of core design is structure D.

Imine bonds have long been regarded as providing an excellent functionality for making a catalyst-free vitrimer because of their easy preparation and the catalyst-free metathesis reaction they enabled.^24−26^ Drawing inspiration from a study of liquid crystals based on an aromatic–imine bond, we adopt this old idea and design new reactive mesogens that possess liquid crystallinity and an intrinsic ability for imine metathesis at the same time.^27^ Because of the rigidity of the aromatic–imine bond, we think it would be interesting to insert it into a mesogenic core to construct a new mesogen molecule. By doing so we can (1) screen through various mesogen cores and find the preferred structure using the fast imine synthesis, and (2) fabricate imine-based xLCEs that are reprocessable owing to the exchangeable imine groups in the mesogenic cores. Our goal is to obtain an imine-based reactive mesogen that has bifunctional acrylate-capped long aliphatic chains as tails and methyl group(s) in the rigid core. The latter is needed to have a low enough nematic transition temperature so that we can obtain stable actuation without entering the temperature range of imine-metathesis onset.^28^ Another important consideration is that the mesogen containing the exchangeable site (the same as the ester group, participating in transesterification in the popular RM257) should not lose the mesogenic power after the exchange (as is the case with RM257). The initial investigation examined four different structures of mesogenic cores as shown in Figure 1. Structure A was ruled out because its starting chemical 2,5-diaminotoluene is prone to oxidation in air and painstaking to handle. Structure B was ruled out because the obtained mesogen has poor solubility in solvents, so the mesogen is difficult to experiment with. Structure C was ruled out because it offers a small aspect ratio and anisotropy to the mesogen. Structure D had its starting chemicals stable in open air. Therefore, structure D was selected and morphed into a reactive mesogen by extending it with aliphatic chains; this mesogen has good solubility in various solvents. We provisionally name this reacting mesogen RM736 on account of its molecular weight of 736 g/mol.

On the basis of the structure of this mesogenic core, we developed a fast and column-free method to synthesize the corresponding imine-based reactive mesogen. Then, we use the “thiol–ene” reaction to connect the mesogen with a thiol-based spacer and a cross-linker to form the imine-based xLCE.^29^ The fabricated xLCE sample displays fast stress–relaxation curves at high temperature due to its catalyst-free imine metathesis between mesogenic cores. The characteristic semisoft stress–strain response of the xLCE at room temperature and its nematic to isotropic transition indicate that the nematic liquid crystalline phase is preserved in the material. We also found that the fast imine metathesis between mesogenic cores at reprocessing temperature does not give noticeable side reactions nor leads to the loss of mesophase, and the xLCE can be realigned to show reversible thermal actuation as a consequence. The only limitation we found in imine-based xLCE is that it can be hydrolyzed in refluxed acidic water, which may restrict its applications from working in a hot, humid environment.

## Experimental Section

### Starting Materials

4-Hydroxybenzaldehyde (98%, Sigma-Merck), 9-bromo-1-nonanol (98%, Alfa Aesar), anhydrous potassium carbonate (99%, Sigma-Merck), tetrabutylammonium bromide (98%, Sigma-Merck), polyethylene glycol 200 (Sigma-Merck), acryloyl chloride (97%, Sigma-Merck), triethylamine (99.5%, Sigma-Merck), and 2,5-dimethyl-1,4-phenylenediamine (98%, TCI) were used in the synthesis of the reacting mesogen. We also used the thiol spacer 2,2′-(ethylenedioxy)diethanethiol (EDDT) (95%, Sigma-Merck), four-functional cross-linker pentaerythritol tetrakis(3-mercaptopropionate) (PETMP) (95%, Sigma-Merck), and catalyst dipropylamine (DPA) (99%, Sigma-Merck).

### Synthesis of *p*-(9-Hydroxynonyloxy)benzaldehyde: Stage a

4-Hydroxybenzaldehyde (1 g, 8.2 mmol), 9-bromo-1-nonanol (1.82 g, 8.2 mmol), anhydrous potassium carbonate (2.3 g, 16.4 mmol), and tetrabutylammonium bromide (0.1 g, 0.3 mmol) were added to polyethylene glycol 200 (1 g, solvent). The mixture was placed at 80 °C until all elements were melted. After homogenization, the paste was radiated in a microwave at 100 W for 4 min while stirring every 30 s. After cooling, 30 mL of deionized (DI) water was added. The insoluble pale-yellow oil below the aqueous layer was extracted using DCM and washed several times with DI water. The organic phase was dried with anhydrous magnesium sulfate and evaporated to afford a pale-yellow solid. The solid was dissolved in large amounts of hot *n*-heptane and recrystallized by rapidly cooling the solution to afford pure needle-like white crystals. Yield: 86%. ^1^H NMR (400 MHz, CDCl_3_): δ 9.88 (s, 1H), 7.83 (m, 2H), 6.99 (m, 2H), 4.04 (t, *J* = 6.5 Hz, 2H), 3.64 (t, *J* = 6.6 Hz, 2H), 1.81 (dt, *J* = 14.7, 6.7 Hz, 2H), 1.56 (q, *J* = 6.8 Hz, 2H), 1.52–1.41 (m, 4H), 1.41–1.33 (m, 2H), 1.25 (s, 4H).

### Synthesis of 9-(4-Formylphenoxy)nonyl Acrylate: Stage b

Under a nitrogen atmosphere, *p*-(9-hydroxynonyloxy)benzaldehyde (8.625 g, 33 mmol) and triethylamine (4.52 mL, 32 mmol) were dissolved in anhydrous THF (60 mL) at 0 °C. Acryloyl chloride (2.65 mL, 33 mmol) was added dropwise, and the reaction was continued for 1 h. After warming to room temperature, the mixture was centrifuged (3 min, 7000 rpm) and the top layer of THF was collected and evaporated to afford a white paste. The paste was dissolved in anhydrous THF again and washed several times with the same method (using centrifugation). The product was obtained in the form of a white solid. Yield: 58%. ^1^H NMR (400 MHz, CDCl_3_): δ 9.88 (s, 1H), 7.82 (m, 2H), 6.99 (m, 2H), 6.40 (dd, *J* = 17.3, 1.5 Hz, 1H), 6.12 (dd, *J* = 17.3, 10.4 Hz, 1H), 5.81 (dd, *J* = 10.4, 1.5, 1H), 4.15 (t, *J* = 6.7 Hz, 2H), 4.04 (t, *J* = 6.5 Hz, 2H), 1.81 (dt, *J* = 14.8, 6.7 Hz, 2H), 1.67 (dq, *J* = 13.8, 6.6 Hz, 4H), 1.47 (dd, *J* = 9.8, 5.1 Hz, 2H), 1.37 (m, 2H), 1.34–1.30 (m, 4H).

### Synthesis of Diacrylate Imine-Based Reactive Mesogen RM736

9-(4-Formylphenoxy)nonyl acrylate (1.29 g, 4 mmol) and 2,5-dimethyl-1,4-phenylenediamine (0.276 g, 2 mmol) were dissolved in anhydrous THF in the presence of activated 3 Å molecular sieves. The dark brown solution was stirred for 1 h at room temperature. After removing the molecular sieves, the solvent was evaporated. The obtained raw product was washed with hot cyclohexane and filtered. After recrystallization from hot cyclohexane, we obtained golden flaky crystals of the desired product. Yield: 62%. ^1^H NMR (400 MHz, CD_2_Cl_2_): δ 8.35 (s, 2H), 7.86 (m, 4H), 6.98 (m, 4H), 6.84 (s, 2H), 6.36 (dd, *J* = 17.3, 1.6 Hz, 2H), 6.12 (dd, *J* = 17.3, 10.4 Hz, 2H), 5.81 (dd, *J* = 10.4, 1.6 Hz, 2H), 4.13 (t, *J* = 6.7 Hz, 4H), 4.03 (t, *J* = 6.6 Hz, 4H), 2.34 (s, 6H), 1.87–1.75 (m, 4H), 1.67 (p, *J* = 7.0 Hz, 4H), 1.48 (dd, *J* = 8.9, 5.1 Hz, 4H), 1.45–1.34 (m, 8H), 1.28 (s, 8H).

^13^C NMR (100 MHz, CD_2_Cl_2_): 166.49, 162.08, 157.99, 148.94, 130.86, 130.58, 130.45, 129.98, 129.09, 119.65, 114.95, 68.62, 64.99, 30.09, 29.82, 29.67, 29.57, 29.00, 26.35, 26.29, 17.62.

Mass spectroscopy (Waters Vion IMS Qtof, mass over charge ratio, for single-proton charge): found *m*/*z* = 737.453; calcd for C_46_H_60_N_2_O_6_ [M + H] = 737.45.

### Synthesis and Reprocessing of Exchangeable xLCE

The imine-based mesogen RM736 (1.75 g), the thiol spacer EDDT (338 mg), and the thiol cross-linker PETMP (151 mg) were dissolved in 0.8 mL of THF under gentle heating. The thiol groups from the cross-linker accounted for 20% of all of the thiol groups added. Dipropylamine catalyst (40 mg) was added, and the reaction was allowed to proceed at 50 °C for 3 h. The resultant gel was dried to yield a yellow-colored xLCE material.

In order to reshape the as-synthesized elastomer into the ideal size and dimensions for subsequent characterization, the as-synthesized exchangeable xLCE was cut into small pieces and hot pressed at 120 °C, under 0.1 MPa, for 2 min. Then, the obtained remolded homogeneous xLCE film was cut into rectangular strips for further tests. The thermal stability of the xLCE at the reprocessing temperature was assessed through ATR-FTIR (Nicolet iS10) over the course of 10 hot-pressing cycles using the ATR spectrum prehot pressing as the reference.

### Characterization

The stress–relaxation experiments were carried out on a dynamic mechanical analysis instrument (TA DMA850). The xLCE sample was equilibrated to four isotropic temperatures (80, 100, 110, and 120 °C) and then rapidly loaded with a constant 2% strain. The stress–relaxation curve was measured, and the temperature-dependent relaxation time, defined as the point when the current stress level decreases to 1/e of the initial stress, was deduced for each temperature. The four relaxation times τ(*T*) were then fitted against the thermal activation Arrhenius law: ln τ(*T*) = const + (*ΔE*)/*k*_B_*T*, where *k*_B_ is the Boltzmann constant, to calculate the activation energy *ΔE* of the bond-exchange reaction.

The stress–strain curves were measured using the same DMA850 instrument. The experiments were conducted at 50 °C in the nematic phase, thus circumventing the issue of sample brittleness at room temperature due to the formation of crystallites (discussed later). To observe the stress plateau due to mesogen rotation under stress during the polydomain–monodomain transition, the xLCE was stretched at three different strain rates (0.03, 0.01, and 0.0033 s^–1^) until sample failure.

The dynamic mechanical (DMA) temperature ramp was conducted on the same DMA850 instrument. The temperature ranged from −20 to 120 °C with the xLCE sample oscillating at 0.1% strain at 1 Hz, and its storage and loss moduli (with tan δ = *G*″/*G*′) were recorded.

Calorimetric signatures of the phase transformations were measured in two ways. A standard differential scanning calorimeter (PerkinElmer DSC4000) was used alongside a modulated differential scanning calorimeter (MDSC TA DSCQ2000) to differentiate the crystallite melting peak from its mesophase transition peaks. Alternating current (ac) (modulated) calorimetry is an advanced technique described in, e.g. refs (30) and (31). In ordinary DSC we used a lower rate of temperature change (5 °C/min) to be closer to the equilibrium phase properties. The MDSC heating profile ranged from −50 to 100 °C at 3 °C/min, and the temperature was modulated at ±1 °C every 60 s. Thermogravimetric analysis (TGA) was performed on a Discovery SDT650 between 80 and 400 °C with a heating rate of 10 °C/min.

Programming of the uniaxial alignment of fully polymerized xLCE samples was done by hanging a weight under the sample and leaving it in a 50 °C oven for 1 h. The sample was then naturally cooled under the same weight to room temperature. The birefringence in the aligned xLCE was verified with polarized optical microscopy (Olympus BX41). Thermal actuation on the aligned xLCE was observed by heating the taut xLCE using an infrared lamp against a black background. Details of the reversible cyclic strain change in actuation were recorded on a DMA850 at a constant load of 10 kPa to the sample before it was cycled between −10 and 140 °C at a rate of 5 °C/min.

## Results and Discussion

**Figure 2:**
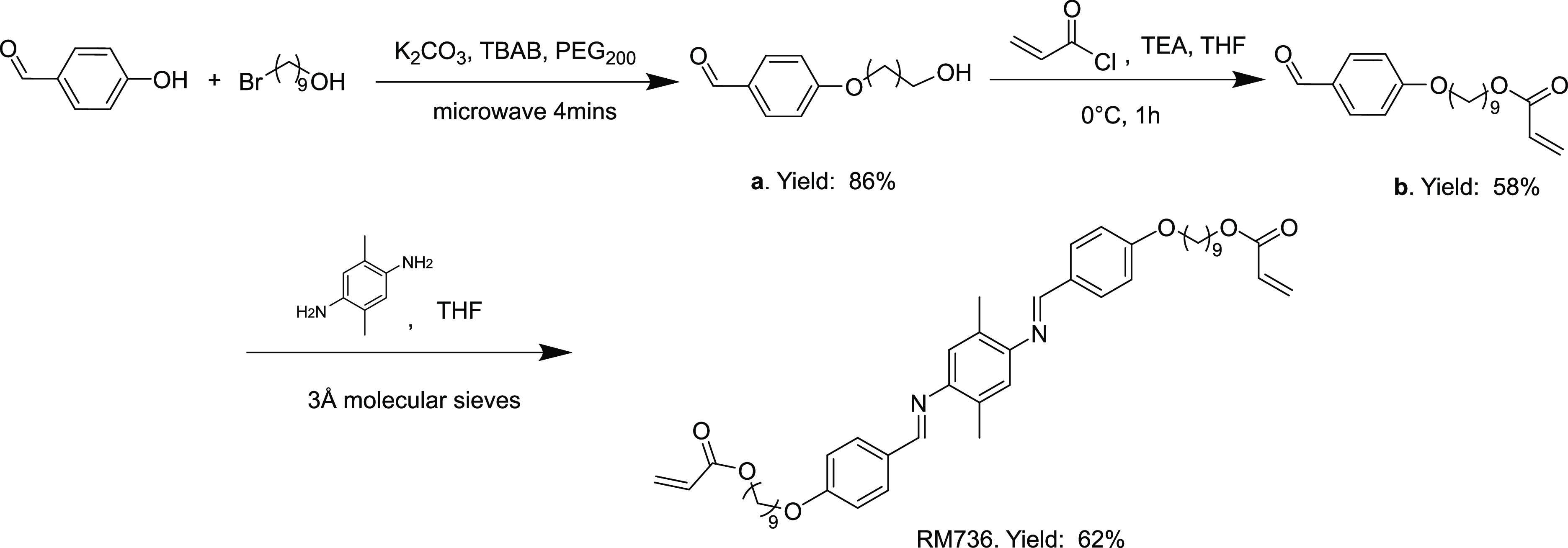
Synthesis scheme for imine-based reactive mesogen. 4-Hydroxybenzaldehyde is extended by a −OH-capped aliphatic chain. Molecule is then acrylated using acryloyl chloride. Resultant benzaldehyde acrylate is condensed with aromatic diamine to generate our imine-base RM736 reactive mesogen.

**Figure 3:**
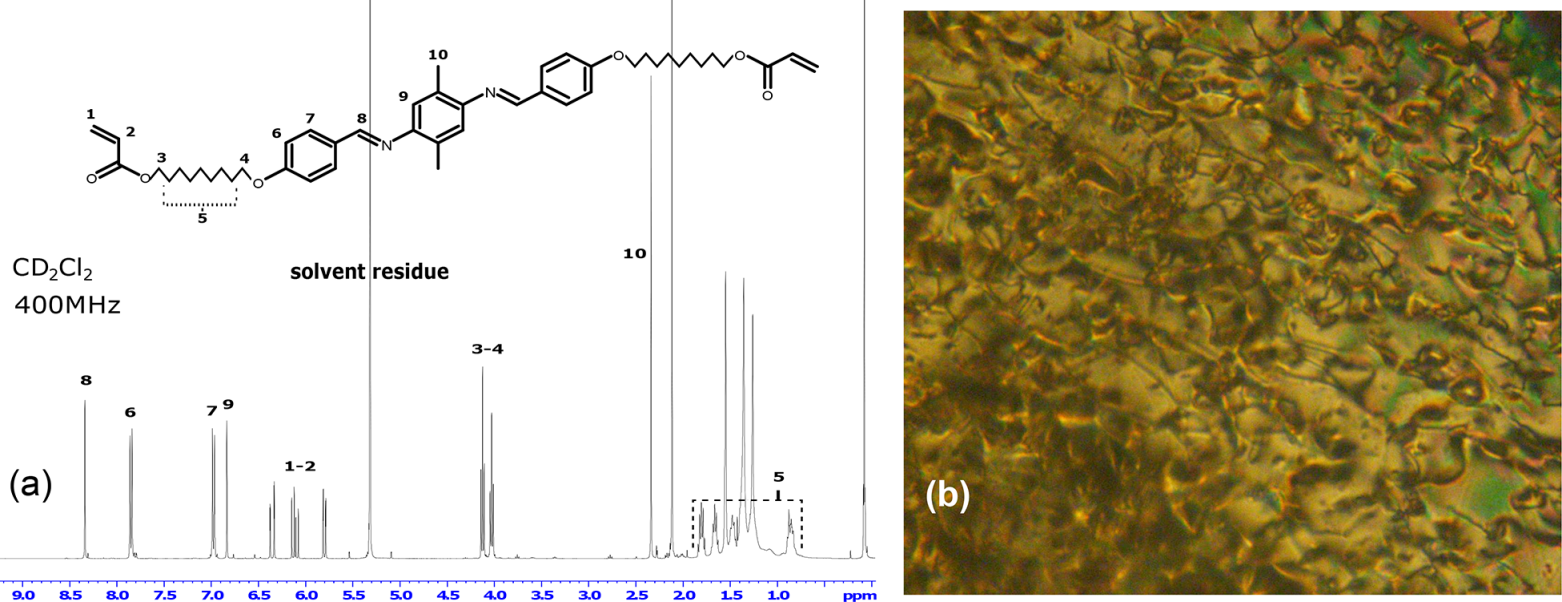
(a) NMR spectrum of the synthesized mesogen molecule with peaks assigned. (b) Mesogen RM736 is heated on a heating stage and observed between crossed polars in a microscope. At 90 °C, the mesogen displays Schlieren texture characteristic of the nematic phase.

A column-free protocol to synthesize an imine-based mesogen has been developed as illustrated in Figure 2. An important first step of the reaction is the alkylation of the benzaldehyde. This step was performed with the assistance of a standard domestic microwave to cut down reaction times. Microwave irradiation has been shown to greatly reduce the Williamson ether synthesis reaction time from hours to minutes with the aid of a phase-transfer catalyst and inorganic base.^32,33^ The choice of 9-bromo-1-nonanol was guided by two factors: on one hand, incorporating longer chains on either side of the mesogen rigid core helps the internal orientational mobility in the final xLCE. On the other hand, in this configuration, 9-bromo-1-nonanol has a lower vapor pressure and less toxicity, enabling it to be safely handled in our household microwave. After a standard procedure of acrylation of the hydroxyl group, the 9-(4-formylphenoxy)nonyl acrylate was condensed with an aromatic diamine in the presence of molecular sieves, and the imine-based mesogen molecule RM736 was quickly generated. Owing to the two protruding methyl groups on the diamine, the mesogen displays good solubility in various organic solvents; we therefore used cyclohexane to recrystallize the product. The NMR spectrum of RM736 presented in Figure 3a shows that the desired molecular structure has been successfully synthesized. We also observe the nematic phase of the mesogen by polarized optical microscopy shown in Figure 3b. The mesogen displays the characteristic nematic Schlieren texture at around 90 °C,^34^ after the RM736 crystals melt. Importantly, the exchangeable imine groups are only present in the mesogen core, so when the bond exchange takes place between one such core and the other, we always have the same amount of mesogenic power in the material. The latter property is due to the symmetry of the core structure: without it, the bond exchange would lead to a variation to the mesogen length.

**Figure 4:**
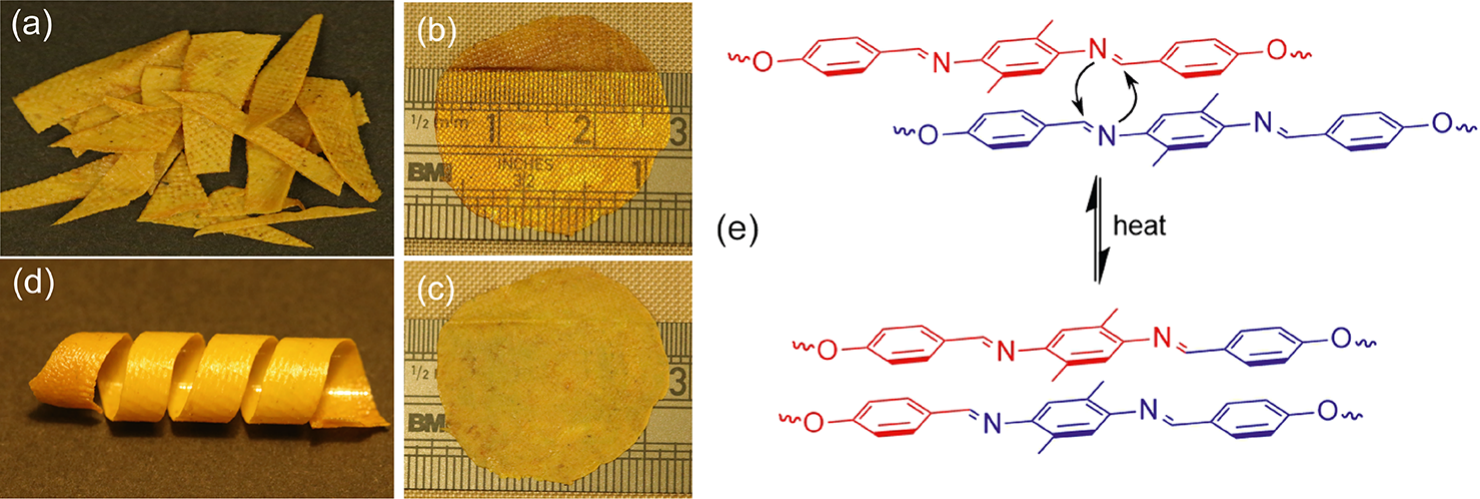
(a) xLCE is cut into pieces and remolded back together into a film (b) after being hot pressed at 120 °C for 2 min. Film is transparent while hot but turns opaque (c) after cooling at ambient temperature. (d) Strip of xLCE is cut from the film and wrapped into a coil. After the coil is left at room temperature for more than 10 min it becomes stiff enough due to microcrystallization, recording this chosen shape; on heating into the isotropic phase, the original flat strip shape is fully recovered in the representation of “shape memory” effect. (e) Illustration of imine metathesis in xLCE. Exchange happens between two different mesogenic cores; the rod-like core structure is preserved after the exchange.

**Figure 5:**
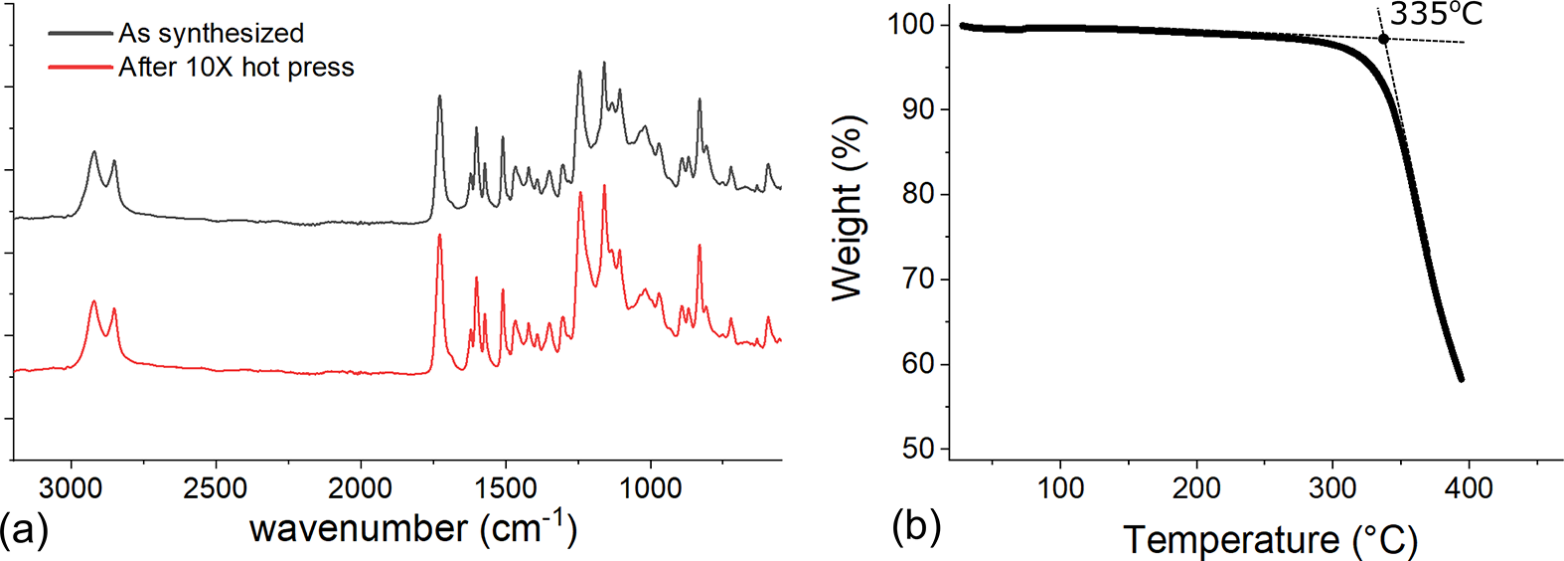
(a) ATR-FTIR spectrum of the xLCE shows no visible change before and after it is remolded 10 times in a hot press, indicating no detrimental side reaction has occurred. (b) TGA thermogram of the xLCE sample shows a degradation onset temperature of 335 °C, a much higher temperature than that needed for imine-based xLCE reprocessing.

The reactive mesogen was polymerized and cross-linked into an imine-based xLCE using a fast “thiol–ene” reaction which is commonly employed to make LCEs.^35,36^ The presence of imine moieties within the xLCE structure opens the door to structural modifications of the thermoset material even postpolymerization, such as self-healing, recycling, and remolding. For instance, the xLCE material obtained was remolded into different dimensions and configurations adapted for further characterization, such as a thin film, using a hot press at 120 °C. We found that our imine-based xLCE can be fully reprocessed within 2 min, as shown in Figure 4a and 4b. The film is transparent right after the hot press because its temperature is still above xLCE isotropic temperature. When cooled (Figure 4c), the film turns opaque owing to the formation of misaligned nematic domains that strongly scatter light.^37^ If the remolded xLCE is left at room temperature for more than 10 min (Figure 4d), the soft xLCE becomes rigid. We assume this is because of the formation of crystalline domains in the long aliphatic spacer chains of our imine-based xLCE. This aspect of our xLCE behavior is the classical shape-memory effect of Lendlein et al.,^38^ where the local crystallinity is helping to record a shape, completely separate from the reversible actuation of aligned LCE that we shall discuss separately below. The dynamic exchange reaction of imine metathesis between two mesogenic cores enables network reconfiguration via plastic flow under stress, as illustrated in Figure 4e. After many cycles of the remolding procedure, the xLCE is shown to be thermally stable as indicated by its unchanged ATR-FTIR spectrum, Figure 5a, before and after the hot pressure remolding and also by thermogravimetric analysis (TGA), Figure 5b. The sample only starts to decompose at a temperature much higher than that required for reprocessing.

The aromatic–imine structure used in our mesogen has a known radical-scavenging capability coming from it is structural similarity to the naturally occurring resveratrol molecule.^39^ Indeed, apart from the Michael reaction used in this report to obtain the imine-based xLCE, we also attempted to photopolymerize the reactive mesogen RM736 through a standard radical-mediated acrylate reaction but never succeeded. We explored radical initiators such as I-651, I-369, I-819, I-2959, or AIBN in order to polymerize and cross-link the reactive monomer, but the sample remained fluid despite UV exposure and/or heat. Such an antioxidative property of the mesogen RM736 perhaps helps explain the remarkable thermal stability seen in our xLCE.

**Figure 6:**
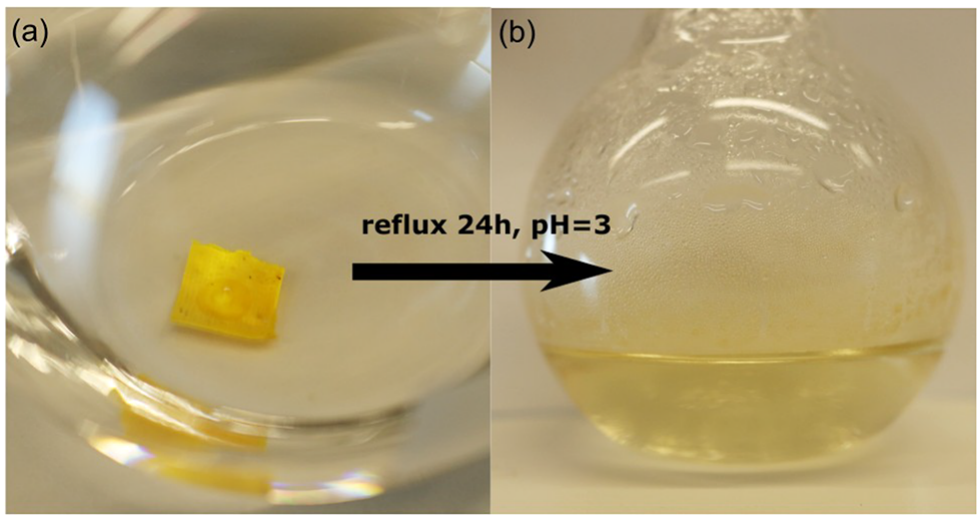
Piece of the xLCE is added to room-temperature deionized water and left for 3 days. It is not hydrolyzed possibly due to a slow water ingression rate into the polymer network. Few drops of glacial acetic acid are then added, bringing the solution to pH = 3. Reflux of the solution for 24 h is shown to be sufficient to completely dissolve the xLCE.

Apart from its radical-scavenging feature, it is well established that the aliphatic imine bonds are prone to hydrolysis in water, and we found it to be also applicable to the imine-based xLCE network, albeit to a lesser degree for our aromatic–imine bonds.^40−42^ Although the imine-based xLCE does not swell in room-temperature water after 3 consecutive days, possibly due to the microcrystalline constraints, stirring it in refluxing acidic water (pH = 3) for 24 h can dissolve the cross-linked network (Figure 6). This evidence suggests that the imine-based xLCE may not be optimal for working in a hot, humid environment. Nevertheless, our rationale shown in this paper in designing mesogens can be extended to make other types of water-resistant mesogen cores that are also intrinsically exchangeable, for example, by utilizing vinylogous urethane bonds.^43^

**Figure 7:**
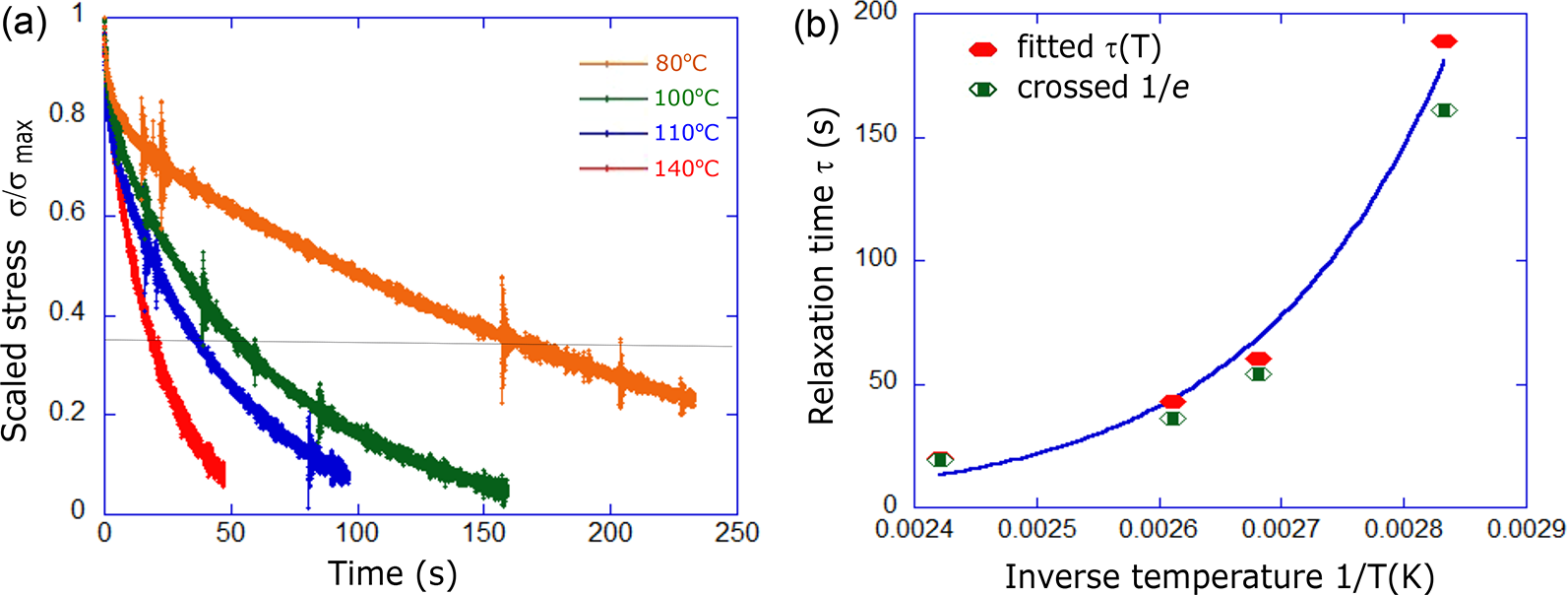
(a) Stress–relaxation curves are measured at four different temperatures and the stress normalized. Temperature-dependent relaxation time τ is taken from each curve in two ways: by fitting an exponential relaxation, or by data crossing the line when σ = (1/*e*)σ_max_. (b) Relaxation time τ(*T*), obtained in two ways, is fitted with the Arrhenius activation law. Activation energy of imine metathesis is calculated to be 54 kJ/mol.

The core property of xLCEs, that is, reconfiguration of the network through selective activation of covalent bond exchange, has a strong dependency on temperature. Though there is no clear threshold for the activation of the bonds when the temperature increases, the kinetics of the phenomenon are such that below a certain temperature the reaction rate is negligible while above a certain temperature the macroscopic reconfiguration of the network occurs increasingly fast, on the scale of hours to minutes. To determine the temperature-dependent bond-exchange rate of the imine-based xLCE, we conducted a series of stress–relaxation tests on the remolded samples with the results shown in Figure 7a. We found imine-based xLCEs to have a short relaxation time (ca. 2 min) at a moderately low temperature of 80 °C. The characteristic relaxation time of each curve at different temperatures was fitted using the Arrhenius thermal activation law, τ = τ_0_ exp[*ΔE*/*k*_B_*T*], as shown in Figure 7b. The “collision time” τ_0_ (or the inverse rate of attempts) was fitted to be τ_0_ = 2 × 10^–6^ s, and the activation energy *ΔE* = 54 kJ/mol. Therefore, the bond-exchange reaction in the imine-based xLCE is a relatively fast process compared to other types of vitrimers,^21,44^ especially considering that no catalyst is required for the imine exchange. For reference, the classical hydroxyl transesterification was reported to have an activation energy of *ΔE* = 90–140 kJ/mol;^10^ the siloxane exchange had *ΔE* = 80–160 kJ/mol.^45^ The borolate transesterification was reported to have a very low *ΔE* = 15 kJ/mol,^46^ although the apparent activation energy can also be affected by the topology of the network matrix.^47,48^

**Figure 8:**
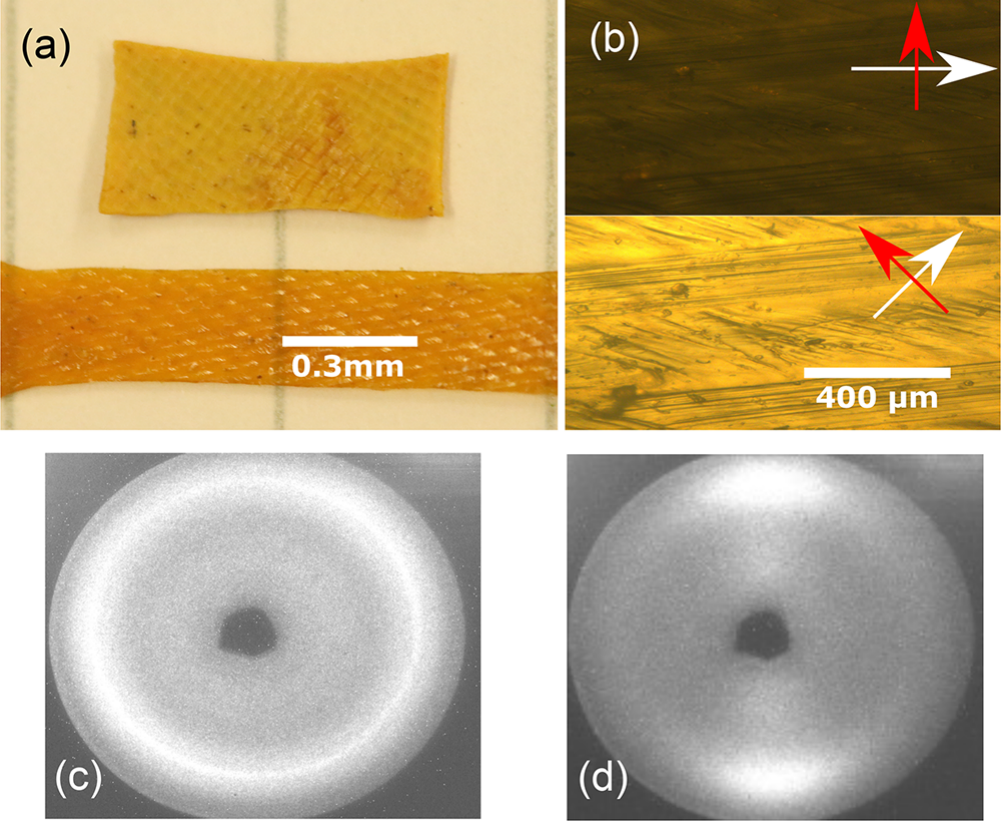
(a) Remolded xLCE can be manually stretched to the transparent state, indicating alignment of their internal nematic director. (b) Images from polarized optical microscopy, aligned and at 45° to the crossed polars. (c and d) X-ray scattering image of the aligned monodomain xLCE, confirming the uniform nematic order and also the lack of any other noticeable structural (smectic or crystalline) features.

As in all of the LCE systems, we found that by manual stretching the remolded, opaque xLCE can be uniformly aligned into a transparent monodomain state, Figure 8a. The uniaxial birefringence is confirmed by rotating the aligned sample between crossed polars, Figure 8b. The parallel X-ray scattering study confirms the transition between a nonaligned and an aligned nematic structure, as illustrated in Figure 8c and 8d. It is surprising that no crystalline features were detected, even though we saw clear evidence of such partial crystallization in the xLCE, among others, leading to the classical shape-memory effect. The answer lies in the calorimetric study of these phase transitions, which follows and allows us to conclude that the degree of crystallinity here is no more than 10%: for such a low fraction of crystallinity, it is not surprising that the relatively crude wide-angle X-ray scattering does not pick up their signal.

**Figure 9:**
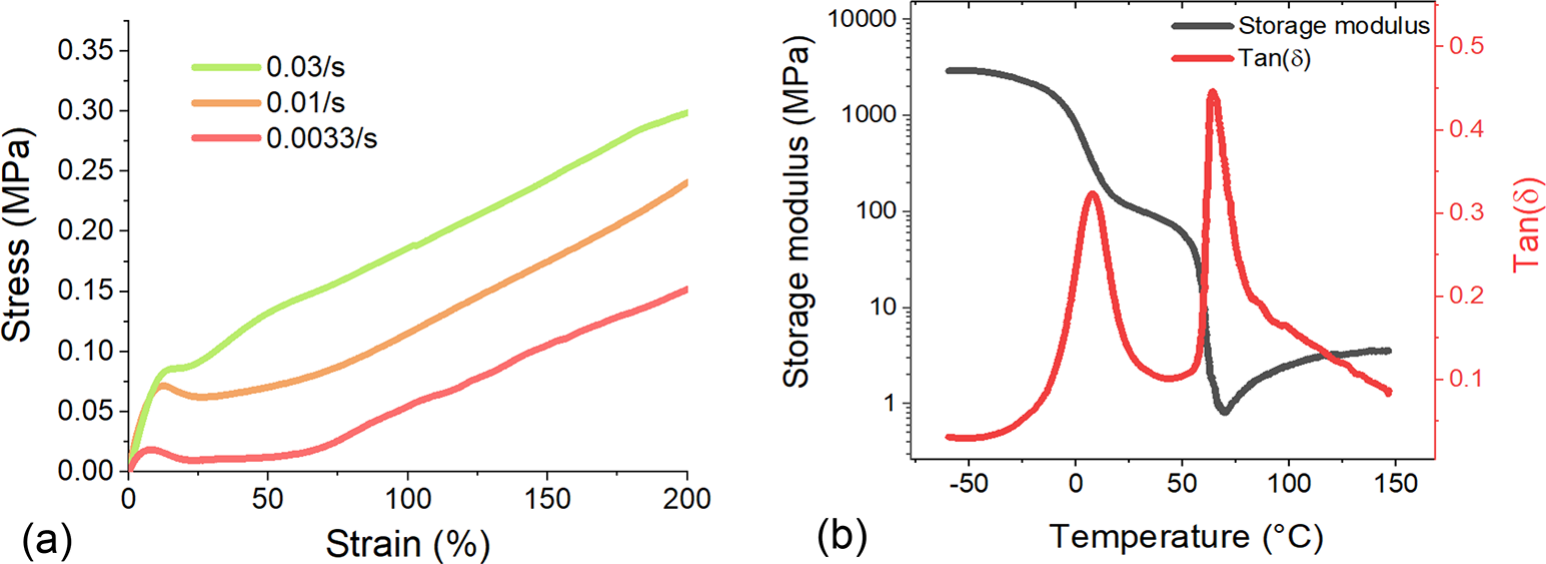
(a) Uniaxial tensile test is carried on the remolded xLCE to show the strain softening as an indication of mesogen rotation. Three strain rates are used in the measurement, and plateau width is clearly shown to be inversely dependent on the strain rate. (b) DMA test is conducted in the xLCE sample at a constant 1 Hz oscillation frequency under a 3 °C/min heating rate. tan(δ) curve shows two peaks which are assigned to the glass and nematic–isotropic transitions. Additional plateau in the storage modulus, below the “soft” nematic transition, is an indication of the sample hardening due to a small fraction of crystallinity, discussed in the text.

The polydomain–monodomain transition in the LCE under a uniaxial stress is revealed in the uniaxial tensile tests shown in the stress–strain data in Figure 9a, which shows stretching xLCE with different strain rates. All strain–stress curves display the characteristic strain-softening plateau, but its width is inversely dependent on the strain rate due to a finite rotation time of realignment of the nematic domains.^49−51^ The presence of the liquid crystalline phase is again indicated in the dynamic mechanical analysis (DMA) test of the xLCE, oscillating at a constant frequency on changing temperature, as shown in Figure 9b. On the basis of the tan δ curve, the glass transition temperature of the sample is around 10 °C, and according to the additional sharp tan δ rise, the “soft” nematic to isotropic temperature is around 70 °C.^52^ In the same test, the linear storage modulus shows a temperature-dependent rubber modulus of around 4 MPa above the isotropic transition and also a clear additional stress plateau of around 100 MPa above the glass transition. This is a much higher modulus than one normally finds in the nematic LCE, even given the reasonably high cross-linking density indicated by the high isotropic modulus. We attribute this xLCE rigidity to the fraction of crystallinity in the sample, which is established after a certain time.

**Figure 10:**
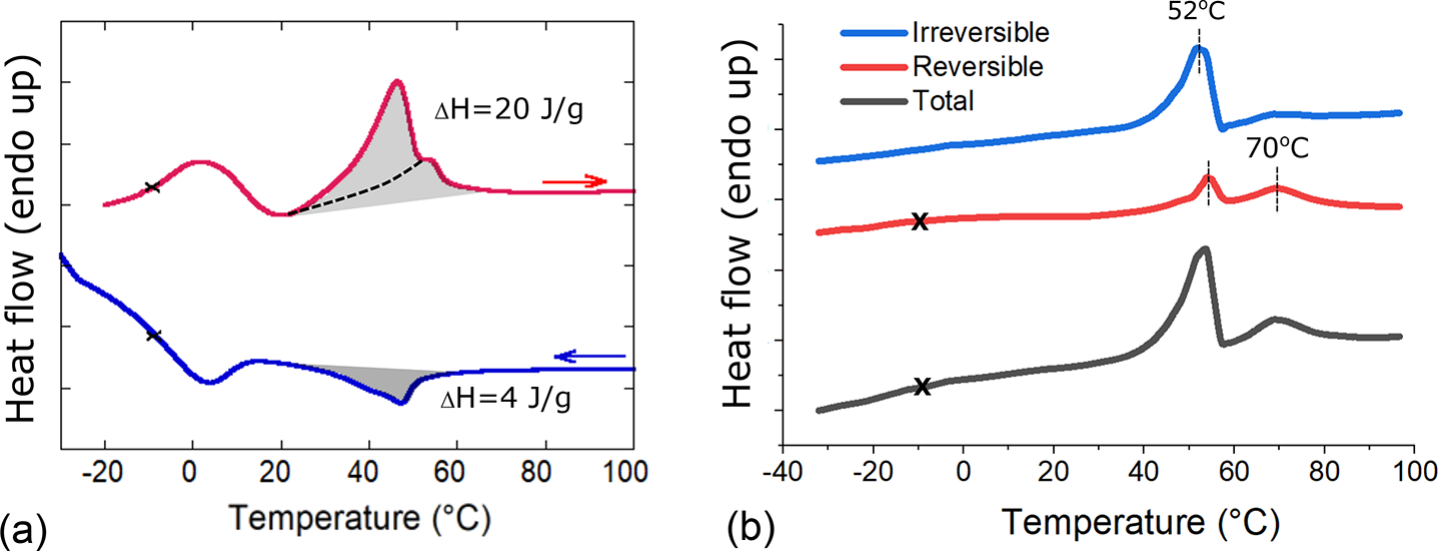
(a) DSC scans show the glass and nematic–isotropic transitions at a rate of heating/cooling of 5 °C/min. On heating, we see the compound transition with a much higher enthalpy, as labeled in the plot: shaded areas illustrate calculation of the transition enthalpy, and dashed line in the heating curve suggests the underlying nematic transition in isolation. On cooling, we only have the nematic transition, because as we have noted in the earlier text it takes some time for the partial crystallinity to establish itself. (b) MDSC scans show the peaks between 52 and 70 °C, which match the ordinary DSC and the DMA data (with a small temperature shift due to a lag in DMA temperature measurement). Complete crystal lamellar melting in xLCE is evident in the irreversible signal in the MDSC test at 52 °C. Nematic–isotropic transitions are shown in the reversible signal curve. In all curves, the glass transition is approximately marked by a cross.

Since our chemistry does not offer the possibility of hydrogen bonding, the first assumption to explain/understand the sample rigidity below the nematic transition could be about a potential smectic phase because LCEs in smectic phase usually have a much higher modulus due to the layer constraints.^16,52−55^ However, the X-ray scattering image in Figure 8d shows no smectic features (we separately examined the small-angle scattering and confirmed no smectic order). Therefore, we turn to the calorimetric study of these phase transitions, Figure 10, illustrating both the DC and the modulated AC calorimetric scanning experiments. The position of the transitions matches the DMA data peaks (with a small inevitable shift due to different temperature measurement), but we find the nature of the isotropic transition to be different from what one expects from the nematic phase. We clearly see the pair of transitions on heating, one of which is nematic, but the other has to be assigned to the melting of partial crystallinity. The enthalpy of this transition is measured to be ca. 20 J/g, which is over 10 times greater than the typical enthalpy of orientational ordering, which is a weak first-order transition^56,57^ (1–5 J/g^58,59^). On cooling we do not see the crystallization, only the nematic peak with the “expected” enthalpy of ca. 4 J/g, which suggests that it takes a long time to form crystalline regions in the elastomer. This is in line with what we observed experimentally, as samples that were initially elastomeric after cooling to room temperature became stiff after about 10 min. If we compare this with the reported enthalpy of crystallization in classical polypropylene (200 J/g^60^) or high-density linear polyethylene (300 J/g^61^), the conclusion is that our xLCE develops a very low degree of crystallinity: certainly less than 10% following this calorimetry data. This would explain why weak crystalline features were not detected in the X-ray studies as well as explain the anomalously high modulus in the nematic phase: the elastomer is effectively a composite with rigid inclusions.^62,63^ Interestingly, a recent study of main-chain LCEs,^64^ where the variable length of the aliphatic spacer was used to control the (low) degree of crystalliity, also did not detect any significant crystalline features in the X-ray results but saw a very high modulus below the nematic–isotropic transition.

In contrast to conventional xLCEs where exchangeable bonds are incorporated in the network through segments external to the mesogen core, imine-based xLCEs are exchanging the bonds within mesogens. We found that the nature of the imine-based xLCE fosters slightly different conditions for the permanent alignment of the mesogens within the network through network dynamic exchange: in the conventional xLCE alignment procedure, samples were heated above their isotropic temperature and allowed to creep under a constant load.^3,4^ During this plastic creep, exchangeable bonds reconfigure the polymer networks to accommodate the external stress into a local uniaxial anisotropy; hence, the network always maintains a nonzero paranematic ordering. As a consequence, this weak anisotropy guides the mesogens to uniformly realign upon entering their nematic phase during subsequent cooling. However, we found that our new imine-based xLCE cannot be aligned above its isotropic temperature, even after a significant amount of creep has occurred. Instead, it is required to be in its nematic phase when subjected to load and creep. We argue that this is caused by the fast imine metathesis between mesogenic cores (so that the locally stretched nonequilibrium network topology is hard to maintain) compounded by the complete disappearance of ordering of mesogens at high temperature (even under load) that the network does not produce any significant anisotropy in the isotropic phase during the induced plastic flow. Therefore, in order to program an aligned imine-based xLCE, the sample was allowed to slowly creep under load in the nematic phase at 50 °C before cooling down.

**Figure 11:**
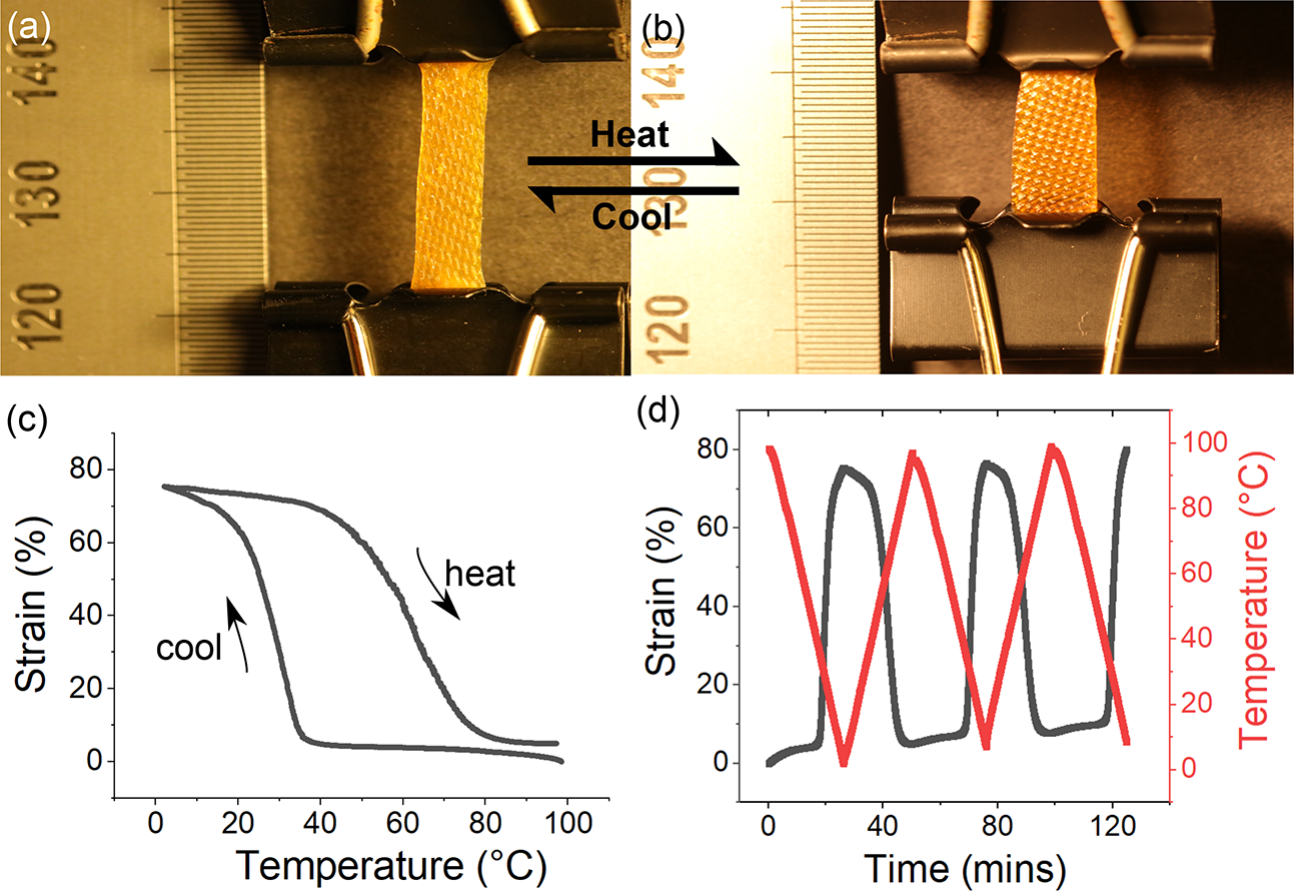
(a and b) Aligned xLCE is capable of reversibly actuating under heat. Sample is lightly stretched by a paper clip and heated using an infrared lamp against a black-absorbing background. (c and d) xLCE sample is loaded with 10 kPa constant stress and heated from 0 to 100 °C at 3 °C/min in a DMA. Strain is calculated based on the equation δ = (*L* – *L*_0_)/*L*, where *L*_0_ is the sample’s initial length at 100 °C. Sample shows a ca. 80% actuation strain in repeated thermal cycling, but creeping is also noticed due to the fast imine exchange reaction.

Following our realignment procedure, the strong uniform birefringence of the xLCE sample was verified under a crossed polarizer and analyzer, as in Figure 8b. Evidence of programmed permanent alignment is also shown in the thermal actuation in Figure 11a and 11b. Thermal actuation is observed in such aligned xLCE by heating a load-free sample under an infrared lamp against a black background. A more detailed actuation test was performed using DMA with the sample under a low constant stress of 10 kPa. In Figure 11c, the xLCE was first equilibrated at 100 °C to eliminate any thermal history, followed by slow cooling until 0 °C, and then heating back up again. As we can see, the sample is able to actuate at 80% strain (using the length at high temperature as a reference). Although this test was carried under a small tensile stress, a non-negligible creep occurred due to the fast imine metathesis in the sample. Such creep is more obvious in Figure 11d, which shows that the imine-based xLCE actuates in repeated steps of thermal cycling.

## Conclusion

In this paper, we designed a new reactive mesogen that combines imine-metathesis chemistry with liquid crystalline properties. A simple, column-free synthesis route was developed that enables relatively fast production of the reactive mesogen molecules which were used to fabricate an imine-based xLCE by “thiol–ene” reaction (three stages, under 1 h each). Due to the presence of the imine groups within the mesogenic cores, capable of undergoing imine metathesis, the fabricated xLCE can be easily reprocessed at 120 °C with no catalyst required. Comparing the ATR-FTIR spectra of the xLCE before and after reprocessing, we concluded that no side reaction happened at this temperature and that the sample remained thermally stable throughout this process. Then, we investigated the imine-based xLCE stress–relaxation behavior at high temperatures and calculated the activation energy of the imine-metathesis reaction to be 54 kJ/mol. Despite the mesogenic cores undergoing quick exchange between themselves at high temperatures, evidence from POM, tensile test, MDSC, and DMA confirms the nematic liquid crystalline ordering in this xLCE. Nonetheless, such a fast metathesis reaction affects the xLCE standard realignment procedure in a way that xLCE needs to be in its nematic phase for the process. The realigned imine-based xLCE materials display a strong birefringence under POM and are capable of large reversible thermal actuation. However, the aligned xLCEs show some degree of creep at the actuating temperature owing to the fast imine metathesis. The imine-based xLCE also can be hydrolyzed in refluxing acidic water. In conclusion, we hope the principles of chemical design shown in this paper inspire further developments of useful mesogens that combine liquid crystallinity with inherent exchangeable bonds.
